# Serum estradiol levels associated with specific gene expression patterns in normal breast tissue and in breast carcinomas

**DOI:** 10.1186/1471-2407-11-332

**Published:** 2011-08-03

**Authors:** Vilde D Haakensen, Trine Bjøro, Torben Lüders, Margit Riis, Ida K Bukholm, Vessela N Kristensen, Melissa A Troester, Marit M Homen, Giske Ursin, Anne-Lise Børresen-Dale, Åslaug Helland

**Affiliations:** 1 Department of Genetics, Institute for Cancer Research, Oslo University Hospital Radiumhospitalet, Oslo, Norway; 2 Institute for Clinical Medicine, Faculty of Medicine, University of Oslo, Oslo, Norway; 3 Department of Oncology, Oslo University Hospital Radiumhospitalet, Oslo, Norway; 4 Deptartment of Medical Biochemistry and Institute of Clinical Biochemistry, Oslo University Hospital Radiumhospitalet, Oslo, Norway; 5 Department of Clinical Molecular Biology, Division of Medicine and Laboratory Sciences, Institute for Clinical Medicine, Akershus University Hospital, University of Oslo, Lørenskog, Norway; 6 Department of Surgery, Akerhus University Hospital, Lørenskog, Norway; 7 Department of Epidemiology and Lineberger Comprehensive Cancer Center, University of North Carolina at Chapel Hill, Chapel Hill, USA; 8 Department of Radiology, University Hospital of North Norway, Tromsø, Norway; 9 Department of Nutrition, School of Medicine, University of Oslo, Oslo, Norway; 10 Department of Preventive Medicine, University of Southern California Keck School of Medicine, Los Angeles, USA; 11 Cancer Registry of Norway, Oslo, Norway

**Keywords:** Serum estradiol, *SCGB3A1*, *HIN1*, *TLN2*, *PTGS1*, *COX1*, *AREG*, *GREB1*, *TFF*, normal breast tissue, gene expression

## Abstract

**Background:**

High serum levels of estradiol are associated with increased risk of postmenopausal breast cancer. Little is known about the gene expression in normal breast tissue in relation to levels of circulating serum estradiol.

**Methods:**

We compared whole genome expression data of breast tissue samples with serum hormone levels using data from 79 healthy women and 64 breast cancer patients. Significance analysis of microarrays (SAM) was used to identify differentially expressed genes and multivariate linear regression was used to identify independent associations.

**Results:**

Six genes (*SCGB3A1, RSPO1, TLN2, SLITRK4, DCLK1, PTGS1*) were found differentially expressed according to serum estradiol levels (FDR = 0). Three of these independently predicted estradiol levels in a multivariate model, as *SCGB3A1 *(*HIN1*) and *TLN2 *were up-regulated and *PTGS1 *(*COX1*) was down-regulated in breast samples from women with high serum estradiol. Serum estradiol, but none of the differentially expressed genes were significantly associated with mammographic density, another strong breast cancer risk factor. In breast carcinomas, expression of *GREB1 *and *AREG *was associated with serum estradiol in all cancers and in the subgroup of estrogen receptor positive cases.

**Conclusions:**

We have identified genes associated with serum estradiol levels in normal breast tissue and in breast carcinomas. *SCGB3A1 *is a suggested tumor suppressor gene that inhibits cell growth and invasion and is methylated and down-regulated in many epithelial cancers. Our findings indicate this gene as an important inhibitor of breast cell proliferation in healthy women with high estradiol levels. In the breast, this gene is expressed in luminal cells only and is methylated in non-BRCA-related breast cancers. The possibility of a carcinogenic contribution of silencing of this gene for luminal, but not basal-like cancers should be further explored. PTGS1 induces prostaglandin E2 (PGE2) production which in turn stimulates aromatase expression and hence increases the local production of estradiol. This is the first report studying such associations in normal breast tissue in humans.

## Background

Influence of estradiol on breast development [1], the menopausal transition [2] and on the breast epithelial cells [3] is widely studied. However, little is known about the effect of serum estradiol on gene expression in the normal breast tissue. For postmenopausal women, high serum estradiol levels are associated with increased risk of breast cancer [4-6]. The results are less conclusive for premenopausal women, but epidemiologic evidence indicates an increased risk from higher exposure to female hormones [7].

In estrogen receptor (ER) positive breast carcinomas, the proliferating tumor cells express ER while in normal breast tissue the proliferating epithelial cells are ER negative (ER-) [8,9]. Both normal and malignant breast epithelial cells are influenced by estradiol but through different mechanisms. In the lack of ER, normal breast epithelial cells receive proliferating paracrine signals from ER+ fibroblasts [3]. The importance of estrogen stimuli in the proliferation of ER+ breast cancer cells is evident from the effect of anti-estrogen treatment. Previously, several studies have identified genes whose expression is regulated by estradiol in breast cancer cell lines. Recently, a study reported an association between serum levels of estradiol and gene expression of trefoil factor 1 *(TFF1)*, growth regulation by estrogen in breast cancer 1 *(GREB1)*, PDZ domain containing 1 *(PDZK1) *and progesterone receptor (PGR) in ER+ breast carcinomas [10]. Functional studies on breast cancer cell lines have described that estradiol induces expression of *c-fos *[11] and that exposure to physiologic doses of estradiol is necessary for malignant transformation [12]. Intratumoral levels of estrogens have also been measured and were found correlated with tumor gene expression of estradiol-metabolizing enzymes and the estrogen receptor gene *(ESR1) *[13] and of proliferation markers [14]. A recent study did, however, conclude that the intratumoral estradiol levels were mainly determined by its binding to ER (associated with ESR1-expression). The intratumoral estradiol levels were not found to be associated with local estradiol production [15]. Serum estradiol levels were found to be associated with local estradiol levels in normal breast tissue of breast cancer patients in a recent study [16]. This strengthens the hypothesis that serum estradiol levels influence the gene expression in breast tissue.

Wilson and colleagues studied the effect of estradiol on normal human breast tissue transplanted into athymic nude mice. They identified a list of genes associated with estradiol treatment, including *TFF1, AREG, SCGB2A2, GREB1 *and *GATA3*. The normal tissues used in the xenografts were from breasts with benign breast disease and from mammoplasty reductions [17].

Studies describing associations between serum estradiol levels and gene expression of normal human breast tissue in its natural milieu are lacking. Knowledge about gene expression changes associated with high serum estradiol may reveal biological mechanisms underlying the increased risk for both elevated mammographic density and for developing breast cancer as seen in women with high estradiol levels. We have identified genes differentially expressed between normal breast tissue samples according to serum estradiol levels. Several genes identified in previous studies using normal breast tissue or breast carcinomas are confirmed, but additional genes were identified making important contributions to our previous knowledge.

## Methods

### Subjects

Two cohorts of women were recruited to the study from different breast diagnostic centers in Norway in the period 2002-2007 as described previously [18]. Exclusion criteria were pregnancy and use of anticoagulant therapy. The first cohort consisted of 120 women referred to the breast diagnostic centers who were cancer-free after further evaluation. These will be referred to as healthy women. Breast biopsies were taken from an area with some mammographic density in the breast contralateral to any suspect lesion. The second cohort consisted of 66 women who were diagnosed with breast cancer. For this cohort, study biopsies were taken from the breast carcinoma after the diagnostic biopsies were obtained. Fourteen gauge needles were used for the biopsies and sampling was guided by ultrasound. The biopsies were either soaked in RNAlater (Ambion, Austin, TX) and sent to the Oslo University Hospital, Radiumhospitalet, before storage at -20°C or directly snap-frozen in liquid nitrogen and stored at -80°C. Based on serum hormone analyses (see below), 57 of the 120 healthy women included were postmenopausal, 43 were premenopausal, 10 were perimenopausal and serum samples were lacking for 10 women. Of the 66 breast cancer patients, 50 were estimated to be postmenopausal, 13 to be premenopausal and 3 to be perimenopausal. All women provided information about height, weight, parity, hormone therapy use and family history of breast cancer and provided a signed informed consent. The study was approved by the regional ethical committee (IRB approval no S-02036).

Three additional datasets were used to explore the regulation of identified genes in breast cancer. One unpublished dataset from the Akershus University Hospital (AHUS), Norway, included normal breast tissue from 42 reduction mammoplasties and both tumor and normal adjacent tissue from 48 breast cancer patients (referred to as the AHUS dataset). Another unpublished dataset from University of North Carolina (UNC), USA, included breast cancer and adjacent normal breast tissue from 55 breast cancer patients (referred to as the UNC dataset). The third dataset is previously published and consists of biopsies from 31 pure ductal carcinoma in situ (DCIS), 36 pure invasive breast cancers and 42 tumours with mixed histology, both DCIS and invasive [19].

### Serum hormone analysis

Serum hormone levels (LH, FSH, prolactin, estradiol, progesterone, SHBG and testosterone) were measured with electrochemiluminescence immunoassays (*ECLIA*) on a Roche Modular E instrument (Roche, Basel, Switzerland) by Department of Medical Biochemistry, Oslo University Hospital, Rikshospitalet. The menopausal status was determined based on serum levels of hormones, age and hormone use. The criteria used can be found in Additional file 1. Biochemically perimenopausal women or women with uncertain menopausal status were excluded from analyses stratified on menopause. These hormone assays are tested through an external quality assessment scheme, Labquality, and the laboratory is accredited according to ISO-ES 17025. Serum estradiol values are given as picograms per milliliter (pg/ml) (pg/ml × 3.67 = pmol/). The functional sensitivity of the estradiol assay was 10.9 pg/ml (40 pmol/l) with a total analytical sensitivity of < 5%.

### Gene expression analysis

RNA extraction and hybridization were performed as previously described [18]. Briefly, RNeasy Mini Protocol (Qiagen, Valencia, CA) was used for RNA extraction. Forty samples (38 from healthy women) were excluded from further analysis due to low RNA amount (< 10 ng) or poor RNA quality assessed by the curves given by Agilent Bioanalyzer (Agilent Technologies, Palo Alto, CA). The analyses were performed before RNA integrity value (RIN) was included as a measure of degradation and samples with poor quality were excluded. Agilent Low RNA input Fluorescent Linear Amplification Kit Protocol was used for amplification and labelling with Cy5 (Amersham Biosciences, Little Chalfont, England) for sample RNA and Cy3 (Amersham Biosciences, Little Chalfont, England) for the reference (Universal Human total RNA (Stratagene, La Jolla, CA)). Labelled RNA was hybridized onto Agilent Human Whole Genome Oligo Microarrays (G4110A) (Agilent Technologies, Santa Clara, CA). Three arrays were excluded due to poor quality leaving data from 79 healthy women and 64 breast cancer patients.

The scanned data was processed in Feature Extraction 9.1.3.1 (Agilent Technologies, Santa Clara, CA). Locally weighted scatterplot smoothing (lowess) was used to normalize the data. The normalized and log2-transformed data was stored in the Stanford Microarray Database (SMD)[20] and retrieved for further analysis. Gene filtering excluded probes with ≥ 20% missing values and probes with less than three arrays being at least 1.6 standard deviation away from the mean. This reduced the dataset from 40791 probes to 9767 for the healthy women and to 10153 for the breast cancer patients. Missing values were imputed in R using the method impute. knn in the library impute [21]. All expression data are available in Gene Expression Omnibus (GEO)(GSE18672).

### Mammographic density

Mammographic density was estimated from digitized craniocaudal mammograms as previously described [18] using the University of Southern California Madena assessment method [22]. First, the total breast area was outlined using a computerized tool and the area was represented as number of pixels. One of the co-authors, GU, identified a region of interest that incorporated all areas of density excluding those representing the pectoralis muscle and scanning artifacts. All densities above a certain threshold were tinted yellow, and the tinted pixles converted to cm^2 ^representing the absolute density and was available for 108 of 120 healthy women. Percent mammographic density is calculated as the absolute density divided by the total breast area and was available for 114 of 120 healthy women. Test-retest reliability was 0.99 for absolute density.

### Statistical Analysis

Quantitative significance analysis of microarrays (SAM) [23,24] was used for analysis of differentially expressed genes, by the library samr in R 2.12.0. Serum estradiol (nmol/L) was used as dependent variable. The distribution of serum levels is skewed and therefore the non-parametric Wilcoxon test-statistic was used. Probes with an FDR < 50% were included for gene ontology analyses.

DAVID Bioinformatics Resources 2008 from the National Institute of Allergy and Infectious Diseases, NIH [25] was used for gene ontology analysis. Functional annotation clustering was applied and the following annotation categories were selected: biological processes, molecular function, cellular compartment and KEGG pathways. We included annotation terms with a p-value (FDR-corrected) of < 0.01 containing between 5 and 500 genes.

For multivariate analysis, linear regression was fitted in R 2.12.0 to identify independent associations. Stepwise selection was performed to determine which variables had an independent contribution to the response variable. In the first step, all variables were included in the model. The variable with the highest p-value was rejected from the model in each step, before the model was refitted. This was repeated until all variables in the model had a p-value smaller than 0.05.

Linear regression was used to determine the independent association between serum estradiol and the differentially expressed genes in healthy women. Age, menopause and current hormone use were included in the model and forced to stay throughout the stepwise selection to correct for confounding by these factors. Linear regression was also fitted in two analyses with mammographic density in healthy women as a dependent variable. In one set of analyses serum hormone levels were included as the independent covariates, and in the other analysis, variables representing gene expression associated with serum estradiol were included as covariates. Epidemiologic covariates, such as age, BMI, parity and use of hormone therapy were included in the mammographic density analyses and forced to stay throughout the stepwise selection to control for potential confounding by these factors.

Tumor subtypes were calculated using the intrinsic subtypes published by Sørlie et al in 2001 [26]. The total gene set was filtered for the intrinsic genes. The correlation between gene expression profiles for the intrinsic genes for each sample with each subtype was calculated. Each sample was assigned to the subtype with which it had the highest correlation. Samples with all correlations < 0.1 were not assigned to any subtype. Two-sided t-tests were used to check for difference in expression for single genes between two categories of variables (eg: pre- and postmenopausal).

## Results

### Gene expression in normal breast tissue according to serum estradiol levels

Genes differentially expressed in normal breast tissue from healthy women according to serum estradiol levels with FDR = 0 are listed in Table 1. The gene ontology terms *extracellular region *and *skeletal system development *were significantly enriched in the top 80 up-regulated genes (FDR < 50%). There were no significant gene ontology terms enriched in the down-regulated genes with FDR < 50 (n = 8), although *response to steroid hormone stimulus *was the most enriched term with three observed genes (prostaglandin-endoperoxide synthase 1 (*PTGS1), ESR1 *and *GATA3*)(Additional file 2).

**Table 1:** Genes significantly differentially expressed in normal breast tissue of healthy women according to serum estradiol.

	Gene Name	*SCGB3A1*	*SLITRK4*	*TLN2*	*DCLK1*	*RSPO1*	*PTGS1*
**A**	Chromosomal location of the gene	5q35.3	Xq27.3	15q15-q21	13q13	1p34.3	9q32-q33.3
	q-value (%) SAM^1)^	0	0	0	0	0	0
	Gene expression in high s-est^2)^						
	(compared with low s-est)	up	up	up	up	up	down

**B**	BC^3) ^vs normal breast tissue (p-value)^4)^	5.00E-15	1.50E-03	2.60E-04	6.00E-04	4.90E-12	0.02
	Gene expression n BC^3)^						
	(compared with normal tissue)	down	down	down	down	down	up
	
	ER+ BC vs normal tissue^5) 4)^	2.20E-13	0.01	1.30E-03	4.20E-03	3.30E-12	0.05
	Gene expression in ER+ BC						
	(compared with normal tissue)	down	down	down	down	down	up
	
	ER- BC vs normal tissue^6) 4) ^	1.10E-04	0.03	0.01	0.01	0.02	0.05
	Gene expression in ER- BC						
	(compared with normal tissue)	down	down	down	down	down	up
	
	Invasive BC vs DCIS^3) 4)^	0.04	0.12	0.01	0.66	0.24	0.001
	Gene expression in invasive BC						
	(compared with DCIS)	down	-	up	-	-	up

A) Q-values and regulation of gene expression from quantitative SAM analysis of gene expression according to serum estradiol. B) Significance testing of difference in gene expression of the genes identified in A) in different sample cohorts.

1) Q-value from SAM of gene expression in normal breast tissue according to serum estradiol

2) s-est = serum estradiol

3) BC = breast cancer

4) P-value from two-sided t-test

5) ER+ BC = estrogen recepor positive breast cancer (n = 53)

6) ER- BC = estrogen recepor negative breast cancer (n = 8)

The genes differentially expressed in normal breast tissue according to serum estradiol with an FDR = 0 (from Table 1) were tested for differential expression between breast cancer tissue and normal breast tissue from healthy women. All six genes were differentially expressed between carcinomas and normal tissue. Interestingly, the expression in breast carcinomas was similar to that in normal tissue from women with lower levels of circulating estradiol and opposite to that found in normal samples from women with higher levels of serum estradiol (Table 1). Comparing the expression of these genes in normal breast tissue with the expression in ER+ and ER- carcinomas respectively revealed similar results (Table 1).

In tumors, *SCGB3A1 *tended to be expressed at a lower level in basal-like tumors compared with all other tumors or compared with luminal A tumors, but this did not reach statistical significance (both p-values = 0.2). However in two other datasets (AHUS and UNC), *SCGB3A1 *was expressed at significantly lower levels in basal-like tumors compared with all other subtypes (p = 0.04 and 0.003 respectively). There was no consistent significant difference in *SCGB3A1 *expression in ER+ and ER- tumors.

Of the six genes differentially expressed according to serum estradiol in normal breast tissue, three were differentially expressed between DCIS and early invasive breast carcinomas based on a previously published dataset [19](Table 1). *SCGB3A1 *was down-regulated in invasive compared with DCIS, whereas talin 2 *(TLN2) *and *PTGS1 *were up-regulated in invasive compared with DCIS.

A linear regression was fitted with all differentially expressed genes as covariates and controlling for age, menopause and current hormone therapy use. After leave-one-out elimination of insignificant covariates, *SCGB3A1, TLN2 *and *PTGS1 *were still significant (Table 2).

**Table 2:** Genes independently associated with serum estradiol in a linear regression model.

Covariate	**Estimate**^**1)**^	Std error	p-value
*SCGB3A1*	0.068	0.025	0.009
*TLN2*	0.142	0.061	0.024
*PTGS1*	-0.145	0.066	0.030
*SLITRK4*	0.086	0.075	0.25^2)^
*RSPO1*	0.045	0.045	0.32^2)^
*DCLK1*	0.023	0.063	0.71^2)^

All genes differentially expressed according to serum estradiol (Table 1) were included. Values shown are corrected for age, menopause and current hormone therapy. After leave-one-out stepwise selection the following covariates remained:

1) Estimate denotes the beta-value corresponding to each covariate in the regression equation.

2) Values for the non-significant genes are from the last model before they were excluded.

### Serum estradiol related to mammographic density in healthy women

Regression analysis in postmenopausal women showed that serum estradiol was independently associated with both absolute and percent mammographic density when controlling for age, BMI and current use of hormone therapy (Table 3). None of the genes differentially expressed in normal breast tissue according to serum estradiol levels were independently associated with mammographic density (data not shown).

**Table 3:** Serum hormones independently associated with mammographic density in linear regression models.

	Absolute density		Percent density	
Covariate	**Estimate**^**1)**^	p-value	Estimate	p-value
Parity	-8.18	0.01	-	-
Serum estradiol	95.55	7.1E-05	51.31	9.3E-03

Values shown are corrected for age, HT and BMI. Through leave-one-out stepwise elimination of covariates, prolactin, SHBG and testosterone were excluded and the following variables remained.

1) Estimate denotes the beta-value corresponding to each covariate in the regression equation.

### Gene expression in breast carcinomas according to serum estradiol levels

In breast carcinomas, quantitative SAM revealed two genes, *AREG *and *GREB1*, as differentially expressed according to serum estradiol levels with FDR = 0 (Table 4). Both genes were up-regulated in samples from women with high serum estradiol (estradiol was used as a continuous response variable in the analysis). Of 16 probes up-regulated in samples from women with high serum estradiol, there were three probes for *TFF3 *and one for *TFF1*, although these did not reach statistical significance (Table 4). No genes were significantly down-regulated according to serum estradiol. In ER+ samples (n = 53), we also found *AREG *and *GREB1 *up-regulated in samples from women with high serum estradiol (FDR = 0), but the *TFF*-genes were not up-regulated. Among the ER- samples (n = 8) there was very little variation in serum estradiol levels and a search for genes differentially expressed according to serum estradiol is not feasible.

**Table 4:** Genes significantly differentially expressed according to serum estradiol levels in breast carcinomas.

	Gene Name	*AREG*	*GREB1*	*TFF3^7)^*	*TFF3^7)^*	*TFF1*
**A**	Chromosomal location	4q13-21	2p25.1	21q22.3	21q22.3	21q22.3
	q-value (%) SAM all tumors^1)^	0	0	20.5	20.5	20.5
	Gene expression in high s-est					
	(compared with low s-est)^2)^	up	up	up	up	up
	
	q-value (%) SAM ER+ BC^1)3)^	0	0	-	-	-
	Gene expression in high s-est					
	(compared with low s-est)^2)^	up	up	-	-	-

**B**	BC^4) ^vs normal breast tissue^5^	0.38	0.18	4.80E-05	2.60E-04	1.20E-07
	Gene expression in BC^4)^					
	(compared with normal)	-	-	up	up	up
	
	ER+ vs ER- BC^4) 5^	0.08	4.80E-08	2.00E-06	2.40E-07	0.002
	Gene expression in ER+ BC					
	(compared with ER- BC^6)^)	up	up	up	up	up

A) Quantitativ SAM analysis for differential expression according to serum estradiol with q-values and direction of regulation indicated. B) Significance testing of difference in gene expression of the genes identified in A) in different sample cohorts.

1) Q-value from SAM of gene expression according to serum estradiol

2) Gene expression in samples from patients with high compared with low serum estradiol

3) ER+ BC = Estrogen receptor positive breast cancer (n = 53)

4) BC = breast cancer

5) P-value from two-sided t-test

6) ER- BC = Estrogen receptor negative breast cancer (n = 8)

7) Two different probes for TFF3 are used

Looking at the expression of these genes in normal breast tissue from healthy women according to serum estradiol, both *AREG *and *GREB1 *are up-regulated in samples from women with high estradiol levels without reaching significance. Comparing the expression of these genes in breast carcinomas and normal breast tissue, neither *AREG *nor *GREB1 *are differentially expressed between normal breast tissue and breast carcinomas. All the probes for *TFF*-genes are, however, significantly down-regulated in normal breast tissue compared with breast carcinomas. All these genes (*AREG, GREB1, TFF1 and TFF3*) were up-regulated in ER+ carcinomas compared to ER- carcinomas (*AREG *was only borderline significant) (Additional file 3).

## Discussion

### Gene expression in normal breast tissue according to serum estradiol levels

We have identified genes differentially expressed according to serum estradiol in normal breast tissue of healthy women.

The genes up-regulated in normal breast tissue under influence of high serum estradiol are enriched for the gene ontology terms *extracellular matrix *and *skeletal system development*. Both ER isoforms α and β are expressed in the stromal cells [27]. The proliferating epithelial cells are not found to be ER α + [8] and most often negative to both ER isoforms [9]. In normal breast tissue, the estrogen-induced epithelial proliferation is, at least partly, caused by paracrine signals from ER+ fibroblasts [3]. The enrichment of gene ontology terms related to extracellular matrix may be linked to the effect of estradiol on the ER+ stromal cells.

Three genes were independently associated with serum estradiol levels in normal breast tissue in a linear regression model after controlling for age, menopause and current hormone therapy. The two genes *SCBG3A1 *and *TLN2 *were positively associated with serum estradiol and *PTGS1 *(*COX1*) negatively.

**Figure 1:**
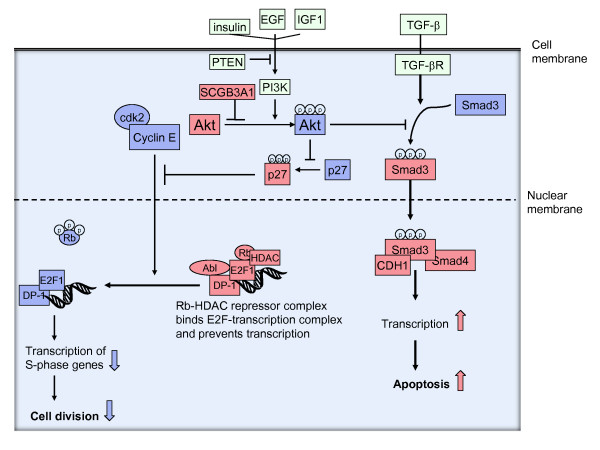
**Simplified illustration of the cellular mechanisms of action of SCGB3A1**. SCGB3A1 inhibits the phosphorylation of Akt leading to reduced cell cycle division and increased apoptosis. Molecules in red are increased/stimulated as result of SCGB3A1-action, whereas molecules in blue are decreased/inhibited.

SCBG3A1 is also called high in normal 1 (HIN1) and is a secretoglobin transcribed in luminal, but not in myoepithelial breast cells and is secreted from the cell [28]. The protein is a tumor suppressor and inhibits cell growth, migration and invasion acting through the AKT-pathway. SCBG3A1 inhibits Akt-phosphorylation, which reduces the Akt-function in promoting cell cycle progression (transition from the G1 to the S-phase) and preventing apoptosis (through inhibition of the TGFβ-pathway) [29] (Figure 1).

The *SCBG3A1 *promoter was found to be hypermethylated with down-regulated expression of the gene in breast carcinomas compared with normal breast tissue, where it is referred to as "high in normal 1" *(HIN1)*[30-32]. Interestingly, the gene is not methylated in BRCA-mutated and BRCA-like breast cancer [32]. Methylation of the gene is suggested to be an early event in non-BRCA-associated breast cancer [33].

We found *SCBG3A1 *down-regulated in basal-like cancers compared to other subtypes. At first glance, this may seem contradictory to the observation that the gene is not methylated in BRCA-like breast cancers. However, Krop and colleagues found that the gene is expressed in luminal epithelial cell lines, but not in myoepithelial cell lines. The reduced expression seen in basal-like cancer could be due to a myoepithelial phenotype arising from a myoepithelial cell of origin or from phenotypic changes acquired during carcinogensis. This could also be linked to the lack of methylation in BRCA-associated breast cancers, which are often basal-like. An a priori low gene expression would make methylation unnecessary. The increased Akt-activity seen in basal-like cancers [34] is consistent with the low levels of SCBG3A1 expression observed in the basal-like cancers in this study leading to increased Akt-phosphorylation and thereby Akt-activity.

The up-regulation of SCGB3A1 in the breasts of women with high serum estradiol protects the breast epithelial cells against uncontrolled proliferation. Women with methylation of the *SCGB3A1*-promoter may be at risk of developing luminal, but not basal-like, breast cancer and a reduction in serum estradiol levels may be protective for these women. Hormone therapy after menopause is associated with receptor positive, but not receptor negative, breast cancer [35]. Our results indicate that the same may be true for circulating estradiol levels in absence of functional SCGB3A1, but this is not yet shown empirically.

**Figure 2:**
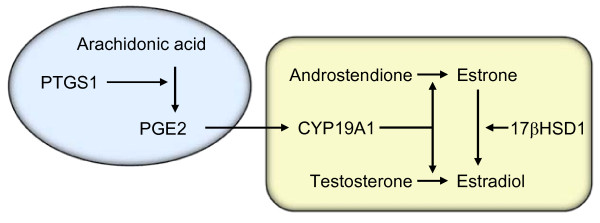
**Schematic illustration of mechanism of action of PTGS1**. PTGS1 induces PGE2-production. PGE2 increases the expression of aromatase (CYP19A1) which in turn converts androgens to estrogens in adipose tissue. 17βHSD1 = 17β-hydroxysteroid dehydrogenase.

*PTGS1 *(prostaglandin-endoperoxide synthase 1) is synonymous with cyclooxygenase 1 (*COX1*) and codes for an enzyme important in prostaglandin production. Studies of normal human adiopocytes have shown that the enzyme induces production of prostaglandin E2 (PGE2) which in turn increases the expression of aromatase (*CYP19A1*) [36]. Aromatase is the enzyme responsible for the last step in the conversion of androgens to estrogens in adipose tissue. Hence, the expression of *PTGS1 *may increase the local production of estradiol (Figure 2). In normal breast tissue, we observed that the expression of *PTGS1 *was lower in samples from women with higher levels of serum estradiol. This may be due to negative feedback. High systemic levels of estradiol make local production unnecessary and PTGS1-induced aromatase production is abolished.

The up-regulation of *PTGS1 *in breast carcinomas compared to normal tissue is expected from current knowledge. Several studies have suggested that *PTGS1 *has a carcinogenic role in different epithelial cancers [37-41]. The gene has also previously been found over-expressed in tumors compared with tumor adjacent normal tissue [42]. There has been large amount of research on *PTGS2 *in relation to cancer, indicating a role in carcinogenesis. The probes for *PTGS2 *were filtered out due to too many missing values and were not included in the analysis. Hence, this study lacks information about the role of PTGS2-expression in relation to serum estradiol levels.

*TLN2 *is less known and less studied than Talin 1 (*TLN1*). Both talins are believed to connect integrins to the actin cytoskeleton and are involved in integrin-associated cell adhesion [43,44]. *TLN2 *is located on chromosome 15q15-21, close to *CYP19A1 *coding for aromatase. A study on aromatase-excess syndrome found that certain minor chromosomal rearrangements may cause cryptic transcription of the *CYP19A1 *gene through the *TLN2*-promoter [45]. We found that *TLN2 *was up-regulated in breasts of healthy women with high levels of serum estradiol. This could indicate an activation of cell adhesion. This gene was the only gene significantly up-regulated according to serum estradiol in normal breast tissue of premenopausal women. The down-regulation observed in breast cancers compared with normal breast tissue indicates a loss of cell adhesion. The expression of the gene is lower in DCIS than in invasive carcinomas, which is contrary to expected, but the data set is small.

A previous study report on the gene expression in normal human breast tissue transplanted into two groups of athymic mice treated with different levels of estradiol [17]. Neither *SCGB3A1*, *TLN2 *nor *PTGS1 *was significantly differentially expressed in their study. They did, however, identify many of the genes found to be significantly differentially expressed according to serum estradiol in breast carcinomas in the current study, such as *AREG*, *GREB1*, *TFF1 *and *TFF3*. Going back to our normal samples, we see that several of their genes (including *AREG*, *GREB1*, *TFF1 *and *TFF3*, *GATA3 *and two *SERPIN*-genes) are differentially expressed in our normal breast tissue, but did not reach statistical significance (Additional file 4).

The differences observed between our study and that of Wilson and colleagues may be due to chance and due to the presence of different residual confounding in the two studies. Wilson and colleagues studied the effects of estradiol treatment, which may act differently upon the breast tissue than endogenous estradiol. Normal human breast tissue transplanted into mice may react differently to varying levels of estradiol than it does in its natural milieu in humans. The genes that were significant in the Wilson-study and differentially expressed but not significant in our study (eg: *AREG*, *GREB1*, *TFF1*, *TFF3 *and *GATA3*) may be associated with serum estradiol levels in normal tissues as well as in tumor tissues where we and others have observed significant associations. Our study is the first study to identify the expression of *SCGB3A1*, *TLN2 *and *PTGS1 *in normal breast tissue to be significantly associated with serum estradiol levels. These findings are biologically reasonable and may have been missed in previous studies due to lack of representative study material.

### Serum estradiol associated with mammographic density in healthy women

Serum estradiol levels were independently associated with mammographic density controlling for age, BMI and current use of hormone therapy, and the magnitude of the association was substantial (Table 3). The high beta-value in the regression equation implies a large magnitude of impact which supports the hypothesis that high serum estradiol levels increases mammographic density with both statistical and biological significance.

### Gene expression in breast carcinomas according to serum estradiol levels

The expression of genes found to be differentially expressed in normal breast tissue according to serum estradiol levels was examined in breast carcinomas. We found that the expression was all opposite of that in normal breast tissue from women with high serum estrogen (Table 1). This may be due to lack of negative feedback of growth regulation in breast tumors. In breast cancer cell lines, estrogen induced up-regulation of positive proliferation regulators and down-regulation of anti-proliferative and pro-apoptotic genes, resulting in a net positive proliferative drive [46]. This is in line with our findings. In normal breast tissue from women with high serum estradiol, *SCGB3A1*, which regulate proliferation negatively, and *TLN2*, which prevents invasion, are up-regulated. *PTGS1*, which induce local production of estradiol-stimulated proliferation, is down-regulated. All three genes are expressed to maintain control and regulation of the epithelial cells. In breast cancers the expression of these genes favors growth, migration and proliferation. This supports the hypothesis that high serum estradiol increases the proliferative pressure in normal breasts, which leads to an activation of mechanisms counter-acting this proliferative pressure. In carcinomas, growth regulation is lost, and these hormone-related growth-promoting mechanisms are turned on.

Interestingly, both *AREG *and *GREB1 *were up-regulated in ER+ breast carcinomas of younger (< 45 years) compared with older (> 70 years) women in a previous publication [47]. The increased expression of these genes was proposed as a mechanism responsible for the observed increase in proliferation seen in the tumors of younger compared with older women [47].

The genes differentially expressed according to serum estradiol levels in tumors confirmed many of the findings from the Dunbier-study of ER+ tumors [10]. The previously published list of genes positively correlated with serum estradiol included TFF-genes and *GREB1*. These genes were also found significant in the analysis of all tumors in this study, although *TFF1 *and *TFF3 *did not reach statistical significance (Table 4). In addition to the previously published genes, we identified the gene *AREG*, an EGFR-ligand essential for breast development, as up-regulated in tumors from patients with high serum estradiol.

GREB1 is previously found to be an important estrogen-induced stimulator of growth in ER+ breast cancer cell lines [48]. AREG binds to and stimulates EGFR and hence epithelial cell growth. The up-regulation of these two genes in breast carcinomas of women with high estradiol levels may indicate a loss of regulation of growth associated with cancer development. This corresponds well with the interpretation of our findings in normal breast tissue referred above and confirms the results indicated by the cell line studies by Frasor and colleagues [46]. These two genes are not differentially expressed between normal breast tissue and breast cancers. Both are, however, higher expressed in ER+ than ER- breast carcinomas.

### Overall strengths and limitations of the study

The currently used method for detection of serum estradiol has a limited sensitivity in the lower serum levels often seen in postmenopausal women. Despite the limited sample size we found several biologically plausible associations. However, due to limited power, there may be other associations that we could not reveal. We have included women with and without hormone therapy in the study. There may be differences in action between endogenous and exogenous estradiol that will not be revealed in this study.

One important strength of this study is the unique material with normal human tissue in its natural mileu, not influenced by an adjacent tumor [49-51] or by an adipose-dominated biology that may bias the study of reduction mammoplasties.

## Conclusions

In conclusion we report a list of genes whose expression is associated with serum estradiol levels. This list includes genes with known relation to estradiol-signaling, mammary proliferation and breast carcinogenesis. All these genes were expressed differently in tumor and normal breast tissue. The gene expression in tumors resembled that in normal breast tissue from women with low serum estradiol. Associations between serum estradiol and the expression in breast carcinomas confirmed previous findings and revealed new associations. The comparison of results between normal breast tissue from healthy women and breast carcinomas indicate the difference in biological impact of estradiol in normal and cancerous breast tissue.

## List of abbreviations

ER: Estrogen receptor; *TFF*: Trefoil factor; *GREB1*: Growth regulation by estrogen in breast cancer 1; *PDZK1*: PDZ domain containing 1; *PGR*: Progesterone receptor; *ESR1*: Estrogen receptor gene; *AREG*: Amphiregulin; *SCGB3A1*: Secretoglobin 3A1; *GATA3*: GATA binding protein 3; DCIS: Ductal carcinoma in situ; MDG: Mammographic density and genetics; AHUS: Akershus University Hospital; UNC: University of North Carolina; LH: luteinizing hormone; FSH: Follicle-stimulating hormone; SHBG: Sex hormone binding globulin; SAM: Significance analysis of microarrays; FDR: False discovery rate; BMI: Body mass index; *PTGS1*: prostaglandin-endoperoxide synthase 1; *TLN2*: Talin 2; *HIN1*: High in normal 1; *COX1*: cyclooxygenase 1.

## Competing interests

The authors declare that they have no competing interests.

## Authors' contributions

VDH assisted in data collection, carried out laboratory work, estimation of mammographic density, statistical analysis, interpretation of results and wrote the paper. TB was responsible for serum hormone analyses. TL contributed to the laboratory work. MR, IKB and MAT assisted in data collection. MMH and VNK designed the study and contributed to data collection. GU designed the study and carried out estimation of mammographic density. ALBD and ÅH designed the study, ensured funding and data collection and interpreted the results. All authors were involved in critically reviewing the report. No medical writers were involved in this paper. All authors have read and approved the final manuscript.

## Pre-publication history

The pre-publication history for this paper can be accessed here:

http://www.biomedcentral.com/1471-2407/11/332/prepub

## Supplementary Material

Additional file 1 **Table S1: Criteria for estimation of menopausal status**. A description of the different criteria used to determine menopausal status. Click here for file

Additional file 2 **Table S2: Gene ontology terms for genes differentially expressed in healthy women according to serum estradiol levels**. A listing of different gene ontology terms for genes differentialle expressed in healthy women dependent on levels of estradiol levels in the serum, with FDR reported. Click here for file

Additional file 3 **Table S3: Genes differentially expressed according to serum estradiol in breast carcinomas and their expression in normal breast tissue. TFF3 represented with two different probes**. Four genes differentially expressed according to serum levels of estradiol, levels in both normal breasts and in breast carcinomas. Click here for file

Additional file 4 **Table S4: Genes differentially expressed according to estradiol treatment in Wilson et al and according to serum estradiol in the current study**. A comparison between a previous published study and this study. Click here for file

## References

[B1] Russo J Russo IH Development of the human breast Maturitas 2004 49 2 15 10.1016/j.maturitas.2004.04.011 15351091

[B2] Burger H The Menopausal Transition -- Endocrinology Journal of Sexual Medicine 2008 5 2266 2273 18624962 10.1111/j.1743-6109.2008.00921.x

[B3] Zhang HZ Bennett JM Smith KT Sunil N Haslam SZ Estrogen mediates mammary epithelial cell proliferation in serum-free culture indirectly via mammary stroma-derived hepatocyte growth factor Endocrinology 2002 143 3427 3434 10.1210/en.2002-220007 12193555

[B4] Key TJ Serum oestradiol and breast cancer risk Endocr Relat Cancer 1999 6 175 180 10.1677/erc.0.0060175 10731106

[B5] Cavalieri EL Stack DE Devanesan PD Todorovic R Dwivedy I Higginbotham S Johansson SL Patil KD Gross ML Gooden JK Molecular origin of cancer: catechol estrogen-3,4-quinones as endogenous tumor initiators Proc Natl Acad Sci USA 1997 94 10937 10942 10.1073/pnas.94.20.10937 9380738 PMC23537

[B6] Hankinson SE Endogenous hormones and risk of breast cancer in postmenopausal women Breast Dis 2005 24 3 15 16917136 10.3233/bd-2006-24102

[B7] Tuma R Mimicking pregnancy to reduce breast cancer risk J Natl Cancer Inst 2010 102 517 518 10.1093/jnci/djq146 20388876

[B8] Clarke RB Howell A Potten CS Anderson E Dissociation between steroid receptor expression and cell proliferation in the human breast Cancer Res 1997 57 4987 4991 9371488

[B9] Saji S Sakaguchi H Andersson S Warner M Gustafsson J Quantitative analysis of estrogen receptor proteins in rat mammary gland Endocrinology 2001 142 3177 3186 10.1210/en.142.7.3177 11416040

[B10] Dunbier AK Anderson H Ghazoui Z Folkerd EJ A'hern R Crowder RJ Hoog J Smith IE Osin P Nerurkar A Relationship between plasma estradiol levels and estrogen-responsive gene expression in estrogen receptor-positive breast cancer in postmenopausal women J Clin Oncol 2010 28 1161 1167 10.1200/JCO.2009.23.9616 20124184 PMC2834467

[B11] Duan R Xie W Li X McDougal A Safe S Estrogen regulation of c-fos gene expression through phosphatidylinositol-3-kinase-dependent activation of serum response factor in MCF-7 breast cancer cells Biochem Biophys Res Commun 2002 294 384 394 10.1016/S0006-291X(02)00499-0 12051724

[B12] Yusuf R Frenkel K Morphologic transformation of human breast epithelial cells MCF-10A: dependence on an oxidative microenvironment and estrogen/epidermal growth factor receptors Cancer Cell Int 2010 10 30 10.1186/1475-2867-10-30 20809984 PMC2944135

[B13] Kristensen VN Sorlie T Geisler J Yoshimura N Linegjaerde OC Glad I Frigessi A Harada N Lonning PE Borresen-Dale AL Effects of anastrozole on the intratumoral gene expression in locally advanced breast cancer J Steroid Biochem Mol Biol 2005 95 105 111 10.1016/j.jsbmb.2005.04.028 16023338

[B14] Geisler J Detre S Berntsen H Ottestad L Lindtjorn B Dowsett M Einstein LP Influence of neoadjuvant anastrozole (Arimidex) on intratumoral estrogen levels and proliferation markers in patients with locally advanced breast cancer Clin Cancer Res 2001 7 1230 1236 11350888

[B15] Haynes BP Straume AH Geisler J A'hern R Helle H Smith IE Lonning PE Dowsett M Intratumoral estrogen disposition in breast cancer Clin Cancer Res 2010 16 1790 1801 10.1158/1078-0432.CCR-09-2481 20215536

[B16] Lonning PE Helle H Duong NK Ekse D Aas T Geisler J Tissue estradiol is selectively elevated in receptor positive breast cancers while tumour estrone is reduced independent of receptor status J Steroid Biochem Mol Biol 2009 117 31 41 10.1016/j.jsbmb.2009.06.005 19591931

[B17] Wilson CL Sims AH Howell A Miller CJ Clarke RB Effects of oestrogen on gene expression in epithelium and stroma of normal human breast tissue Endocr Relat Cancer 2006 13 617 628 10.1677/erc.1.01165 16728587

[B18] Haakensen VD Biong M Lingjaerde OC Holmen MM Frantzen JO Chen Y Navjord D Romundstad L Luders T Bukholm IK Expression levels of uridine 5'-diphospho-glucuronosyltransferase genes in breast tissue from healthy women are associated with mammographic density Breast Cancer Res 2010 12 R65 10.1186/bcr2632 20799965 PMC2949660

[B19] Muggerud AA Hallett M Johnsen H Kleivi K Zhou W Tahmasebpoor S Amini RM Botling J Borresen-Dale AL Sorlie T Molecular diversity in ductal carcinoma in situ (DCIS) and early invasive breast cancer Mol Oncol 2010 4 357 368 10.1016/j.molonc.2010.06.007 20663721 PMC5527914

[B20] Stanford Microarray Database http://genome-www5.stanford.edu/

[B21] R library impute.knn http://rss.acs.unt.edu/Rdoc/library/impute/html/impute.knn.html

[B22] Ursin G Astrahan MA Salane M Parisky YR Pearce JG Daniels JR Pike MC Spicer DV The detection of changes in mammographic densities Cancer Epidemiol Biomarkers Prev 1998 7 43 47 9456242

[B23] Tusher VG Tibshirani R Chu G Significance analysis of microarrays applied to the ionizing radiation response Proc Natl Acad Sci USA 2001 98 5116 5121 10.1073/pnas.091062498 11309499 PMC33173

[B24] Significance Analysis of Microarrays http://www-stat.stanford.edu/~tibs/SAM/

[B25] DAVID Bioinformatics Resources 6.7 http://david.abcc.ncifcrf.gov/

[B26] Sorlie T Perou CM Tibshirani R Aas T Geisler S Johnsen H Hastie T Eisen MB van de RM Jeffrey SS Gene expression patterns of breast carcinomas distinguish tumor subclasses with clinical implications Proc Natl Acad Sci USA 2001 98 10869 10874 10.1073/pnas.191367098 11553815 PMC58566

[B27] Cheng G Li Y Omoto Y Wang Y Berg T Nord M Vihko P Warner M Piao YS Gustafsson JA Differential regulation of estrogen receptor (ER)alpha and ERbeta in primate mammary gland J Clin Endocrinol Metab 2005 90 435 444 15507513 10.1210/jc.2004-0861

[B28] Krop IE Sgroi D Porter DA Lunetta KL LeVangie R Seth P Kaelin CM Rhei E Bosenberg M Schnitt S HIN-1, a putative cytokine highly expressed in normal but not cancerous mammary epithelial cells Proc Natl Acad Sci USA 2001 98 9796 9801 10.1073/pnas.171138398 11481438 PMC55532

[B29] Krop I Parker MT Bloushtain-Qimron N Porter D Gelman R Sasaki H Maurer M Terry MB Parsons R Polyak K HIN-1, an inhibitor of cell growth, invasion, and AKT activation Cancer Res 2005 65 9659 9669 10.1158/0008-5472.CAN-05-1663 16266985

[B30] Park SY Kwon HJ Lee HE Ryu HS Kim SW Kim JH Kim IA Jung N Cho NY Kang GH Promoter CpG island hypermethylation during breast cancer progression Virchows Arch 2010 10.1007/s00428-010-1013-6 21120523

[B31] Krop I Player A Tablante A Taylor-Parker M Lahti-Domenici J Fukuoka J Batra SK Papadopoulos N Richards WG Sugarbaker DJ Frequent HIN-1 promoter methylation and lack of expression in multiple human tumor types Mol Cancer Res 2004 2 489 494 15383627

[B32] Krop I Maguire P Lahti-Domenici J Lodeiro G Richardson A Johannsdottir HK Nevanlinna H Borg A Gelman R Barkardottir RB Lack of HIN-1 methylation in BRCA1-linked and "BRCA1-like" breast tumors Cancer Res 2003 63 2024 2027 12727813

[B33] Vasilatos SN Broadwater G Barry WT Baker JC Jr Lem S Dietze EC Bean GR Bryson AD Pilie PG Goldenberg V CpG island tumor suppressor promoter methylation in non-BRCA-associated early mammary carcinogenesis Cancer Epidemiol Biomarkers Prev 2009 18 901 914 10.1158/1055-9965.EPI-08-0875 19258476 PMC2667866

[B34] Moulder SL Does the PI3K Pathway Play a Role in Basal Breast Cancer? Clin Breast Cancer 2010 10 S66 S71 10.3816/CBC.2010.s.014 21115424

[B35] Rosenberg LU Magnusson C Lindstrom E Wedren S Hall P Dickman PW Menopausal hormone therapy and other breast cancer risk factors in relation to the risk of different histological subtypes of breast cancer: a case-control study Breast Cancer Res 2006 8 R11 10.1186/bcr1378 16507159 PMC1413980

[B36] Zhao Y Agarwal VR Mendelson CR Simpson ER Estrogen biosynthesis proximal to a breast tumor is stimulated by PGE2 via cyclic AMP, leading to activation of promoter II of the CYP19 (aromatase) gene Endocrinology 1996 137 5739 5742 10.1210/en.137.12.5739 8940410

[B37] Kino Y Kojima F Kiguchi K Igarashi R Ishizuka B Kawai S Prostaglandin E2 production in ovarian cancer cell lines is regulated by cyclooxygenase-1, not cyclooxygenase-2 Prostaglandins Leukot Essent Fatty Acids 2005 73 103 111 10.1016/j.plefa.2005.04.014 15963707

[B38] Daikoku T Wang D Tranguch S Morrow JD Orsulic S DuBois RN Dey SK Cyclooxygenase-1 is a potential target for prevention and treatment of ovarian epithelial cancer Cancer Res 2005 65 3735 3744 10.1158/0008-5472.CAN-04-3814 15867369 PMC2584020

[B39] Chulada PC Thompson MB Mahler JF Doyle CM Gaul BW Lee C Tiano HF Morham SG Smithies O Langenbach R Genetic disruption of Ptgs-1, as well as Ptgs-2, reduces intestinal tumorigenesis in Min mice Cancer Res 2000 60 4705 4708 10987272

[B40] Frank B Hoffmeister M Klopp N Llig T Chang-Claude J Brenner H Polymorphisms in inflammatory pathway genes and their association with colorectal cancer risk Int J Cancer 2010 10.1002/ijc.25299 21351261

[B41] Androulidaki A Dermitzaki E Venihaki M Karagianni E Rassouli O Andreakou E Stournaras C Margioris AN Tsatsanis C Corticotropin Releasing Factor promotes breast cancer cell motility and invasiveness Mol Cancer 2009 8 30 10.1186/1476-4598-8-30 19490624 PMC2697132

[B42] Hwang D Scollard D Byrne J Levine E Expression of cyclooxygenase-1 and cyclooxygenase-2 in human breast cancer J Natl Cancer Inst 1998 90 455 460 10.1093/jnci/90.6.455 9521170

[B43] Debrand E El JY Spence L Bate N Praekelt U Pritchard CA Monkley SJ Critchley DR Talin 2 is a large and complex gene encoding multiple transcripts and protein isoforms FEBS J 2009 276 1610 1628 10.1111/j.1742-4658.2009.06893.x 19220457 PMC2702505

[B44] Conti FJ Monkley SJ Wood MR Critchley DR Muller U Talin 1 and 2 are required for myoblast fusion, sarcomere assembly and the maintenance of myotendinous junctions Development 2009 136 3597 3606 10.1242/dev.035857 19793892 PMC2761109

[B45] Demura M Martin RM Shozu M Sebastian S Takayama K Hsu WT Schultz RA Neely K Bryant M Mendonca BB Regional rearrangements in chromosome 15q21 cause formation of cryptic promoters for the CYP19 (aromatase) gene Hum Mol Genet 2007 16 2529 2541 10.1093/hmg/ddm145 17584767

[B46] Frasor J Danes JM Komm B Chang KC Lyttle CR Katzenellenbogen BS Profiling of estrogen up- and down-regulated gene expression in human breast cancer cells: insights into gene networks and pathways underlying estrogenic control of proliferation and cell phenotype Endocrinology 2003 144 4562 4574 10.1210/en.2003-0567 12959972

[B47] Yau C Fedele V Roydasgupta R Fridlyand J Hubbard A Gray JW Chew K Dairkee SH Moore DH Schittulli F Aging impacts transcriptomes but not genomes of hormone-dependent breast cancers Breast Cancer Res 2007 9 R59 10.1186/bcr1765 17850661 PMC2216076

[B48] Rae JM Johnson MD Scheys JO Cordero KE Larios JM Lippman ME GREB 1 is a critical regulator of hormone dependent breast cancer growth Breast Cancer Res Treat 2005 92 141 149 10.1007/s10549-005-1483-4 15986123

[B49] Heaphy C Griffith J Bisoffi M Mammary field cancerization: molecular evidence and clinical importance Breast Cancer Research and Treatment 2009 118 229 239 10.1007/s10549-009-0504-0 19685287

[B50] Graham K de las MA Tripathi A King C Kavanah M Mendez J Stone M Slama J Miller M Antoine G Gene expression in histologically normal epithelium from breast cancer patients and from cancer-free prophylactic mastectomy patients shares a similar profile Br J Cancer 2010 102 1284 1293 10.1038/sj.bjc.6605576 20197764 PMC2855998

[B51] Troester MA Lee MH Carter M Fan C Cowan DW Perez ER Pirone JR Perou CM Jerry DJ Schneider SS Activation of host wound responses in breast cancer microenvironment Clin Cancer Res 2009 15 7020 7028 10.1158/1078-0432.CCR-09-1126 19887484 PMC2783932

