# Supplementary material for: Serum estradiol levels associated with specific gene expression patterns in normal breast tissue and in breast carcinomas
Source: BMC Cancer. 2011 Aug 3;11:332. doi: 10.1186/1471-2407-11-332 (PMC3163631; doi:10.1186/1471-2407-11-332)
Supplement: Additional file 1 — Table S1: Criteria for estimation of menopausal status. A description of the different criteria used to determine menopausal status. [file 1471-2407-11-332-S1.DOC]

**Additional file 1:**

**Table S1:** Criteria for estimation of menopausal status.

| FSH>20 | LH>15 | FSH>LH | s-est^1)^ <27.3 | menopausal status | criteria |
| --- | --- | --- | --- | --- | --- |
| 1 | 1 | 1 | 1 | post |  |
| 1 | 1 | 1 | 0 | post | s-est<82^2)^ |
| 1 | 1 | 1 | 0 | peri | s-est>82^2)^ |
| 1 | 0 | 1 | 1 | post |  |
| 1 | 1 | 0 | 0 | peri | s-est>82^2)^ without HT^3)^ |
| 1 | 1 | 0 | 0 | post | s-est <82^2)^ or HT |
| 1 | 0 | 1 | 0 | post | s-est >82^2)^ without HT |
| 1 | 0 | 1 | 0 | peri | s-est <82^2)^ or HT |
| 0 | 0 | 1 | 1 | peri | FSH>15 |
| 0 | 0 | 1 | 1 | pre | FSH<15^4)^ |
| 0 | 1 | 0 | 0 | pre | Age>50=peri |
| 0 | 0 | 1 | 0 | peri | s-est <82^2)^ |
| 0 | 0 | 1 | 0 | pre | s-est >82^2)^ |
| 0 | 0 | 0 | 0 | pre |  |
| Any | Any | Any | Any | post/peri | HT-use |

1) serum estradiol in pg/ml (= 0.1 nmol/l)

2) 82 pg/ml = 0.3 nmol/l

3) HT: hormone therapy

4) Day 1-3 in the menstrual cycle, estradiol is often low (<0.1) with a slight increase in FSH (<15) and no increase in LH.
