# Supplementary material for: Serum estradiol levels associated with specific gene expression patterns in normal breast tissue and in breast carcinomas
Source: BMC Cancer. 2011 Aug 3;11:332. doi: 10.1186/1471-2407-11-332 (PMC3163631; doi:10.1186/1471-2407-11-332)
Supplement: Additional file 2 — Table S2: Gene ontology terms for genes differentially expressed in healthy women according to serum estradiol levels. A listing of different gene ontology terms for genes differentialle expressed in healthy women dependent on levels of estradiol levels in the serum, with FDR reported. [file 1471-2407-11-332-S2.DOC]

**Additional file 2:**

**Table S2**: Gene ontology terms for genes differentially expressed in healthy women according to serum estradiol levels.

| Up-regulated genes (FDR<50) |  |  |
| --- | --- | --- |
| Gene ontology term | No of genes | FDR |
| Extracellular region | 43 | 0.008 |
| Skeletal system development | 45 | 0.01 |
| Tube development | 45 | 1.1 |
| Extracellular matrix | 43 | 0.6 |
|  |  |  |
| Down-regulated genes (FDR<50) |  |  |
| Gene ontology term | No of genes | FDR |
| Response to seroid hormone stimulus | 3 | 2.1 |
