# Supplementary material for: Serum estradiol levels associated with specific gene expression patterns in normal breast tissue and in breast carcinomas
Source: BMC Cancer. 2011 Aug 3;11:332. doi: 10.1186/1471-2407-11-332 (PMC3163631; doi:10.1186/1471-2407-11-332)
Supplement: Additional file 3 — Table S3: Genes differentially expressed according to serum estradiol in breast carcinomas and their expression in normal breast tissue. TFF3 represented with two different probes. Four genes differentially expressed according to serum levels of estradiol, levels in both normal breasts and in breast carcinomas. [file 1471-2407-11-332-S3.DOC]

**Additional file 3**

**Table S3:** Genes differentially expressed according to serum estradiol in breast carcinomas and their expression in normal breast tissue. TFF3 represented with two different probes.

|  | ***AREG*** | ***GREB1*** | ***TFF3*** | ***TFF3*** | ***TFF1*** |
| --- | --- | --- | --- | --- | --- |
| Agilent probe ID | A_23_P259071 | A_23_P329768 | A_23_P257296 | A_23_P393099 | A_24_P322771 |
| p-value (tumor vs normal)^1)^ | 0.38 | 0.18 | 4.8E-05 | 2.6E-04 | 1.2E-07 |
| Mean tumor | -0.61 | -1.02 | 2.74 | 2.65 | -1.13 |
| Mean normal | -0.35 | -1.29 | 1.45 | 1.44 | -2.76 |
| p-value (ER+ vs ER-)^1)^ | 0.08 | 4.8E-08 | 2.0E-06 | 2.4E-07 | 2.3E-03 |
| Mean ER+ tumors (n=53) | -0.44 | -0.60 | 3.21 | 3.19 | -0.81 |
| Mean ER- tumors (n=8) | -1.54 | -3.17 | -0.17 | -0.51 | -3.32 |
| q-value (%) SAM normal tissue according to serum-estradiol^2)^ | 52.9 | 28.4 | 52.9 | 39.5 | 75.3 |

1. Two-sided t-test
2. Q-value for genes up-regulated in samples from women with high serum estradiol (SAM on samples from normal breast tissue according to serum estradiol).
