# Supplementary material for: Serum estradiol levels associated with specific gene expression patterns in normal breast tissue and in breast carcinomas
Source: BMC Cancer. 2011 Aug 3;11:332. doi: 10.1186/1471-2407-11-332 (PMC3163631; doi:10.1186/1471-2407-11-332)
Supplement: Additional file 4 — Table S4: Genes differentially expressed according to estradiol treatment in Wilson et al and according to serum estradiol in the current study. A comparison between a previous published study and this study. [file 1471-2407-11-332-S4.DOC]

**Additional file 4**

**Table S4:** Genes differentially expressed according to estradiol treatment in Wilson et al and according to serum estradiol in the current study

| Genes differentially expressed between mice treated and not treated with estradiol in Wilson et al, 2006 | | Quantitative SAM according to serum estradiol levels in the current study. Gene expression in high serum estradiol. | | |
| --- | --- | --- | --- | --- |
| Symbol | gene experession in estradiol-treated mice | Normal breast tisssue | Breast carcinomas | ER+ breast carcinomas |
| TFF1 | up | up | up | - |
| MYBPC1 | up | up | - | - |
| AREG | up | up | up | up |
| SCGB2D2 | up | - | - | - |
| TFF3 | up | up | up | - |
| SCGB2A2 | up | - | - | - |
| GREB1 | up | up | up | up |
| SERPINA1 | up | up | - | - |
| C1orf34 | up | up | - | - |
| PIP | up | - | down | down |
| AGR2 | up | - | - | down |
| SERPINA3 | up | up | - | - |
| PRR4 | up | - | - | - |
| HBE1 | up | - | - | - |
| EEF1A2 | up | - | - | - |
| DSU | up | - | - | - |
| MYB | up | - | - | - |
| AZGP1 | up | - | - | down |
| TACSTD1 | up | - | - | down |
| KRT19 | up | - | down | down |
| CELSR2 | up | - | - | - |
| FXYD3 | up | - | - | - |
| XBP1 | up | - | - | down |
| PRG4 | up | - | down | down |
| MMP | up | - | - | - |
| MMP12 | down | - | down | down |
| ME1 | down | up | down | down |
| CXCL11 | down | - | - | - |
| COL6A | down | - | - | - |
| CXCL11 | down | - | - | - |
| GATA3 | down | down | - | - |
| HSPG2 | down | - | - | - |
| CCl2 | down | - | down | down |
| EMILIN | down | - | - | - |
| CXCL10 | down | - | - | - |
| Tabls S4 cont  Genes differentially expressed between mice treated and not treated with estradiol in Wilson et al, 2006 | | Quantitative SAM according to serum estradiol levels. Gene expression in high serum estradiol - | | |
| Symbol | gene expression in treated mice | Normal breast tisssue | Breast cancers | ER+ breast cancers |
| RARRES1 | down | up | down | - |
| S100A8 | down | - | down | down |
| IGJ | down | up | - | - |
| CXCL9 | down | - | down | down |
| FN1 | down | - | - | - |
| DPT | down | - | down | - |
| RGS5 | down | - | down | down |
| CCL19 | down | - | down | down |
| DPT | down | - | down | - |
| TPSAB1 | down | - | - | - |
| CSPG2 | down | up | - | - |
| ENPEP | down | - | - | - |
| SERPINH1 | down | up | - | - |
| RGS5 | down | - | down | down |
| IL8 | down | - | - | - |
| IGHA1 | down | - | down | - |
| BGN | down | - | - | - |
